# Influence of Maternal Care on Behavioural Development of Domestic Dogs (*Canis Familiaris*) Living in a Home Environment

**DOI:** 10.3390/ani7120093

**Published:** 2017-12-05

**Authors:** Giovanna Guardini, Jon Bowen, Chiara Mariti, Jaume Fatjó, Claudio Sighieri, Angelo Gazzano

**Affiliations:** 1 Department of Veterinary Sciences, University of Pisa, Viale delle Piagge, 2, 56124 Pisa, Italy; cmariti@vet.unipi.it (C.M.); csighier@vet.unipi.it (C.S.); agazzano@vet.unipi.it (A.G.); 2 Queen Mother Hospital for Small Animals, Royal Veterinary College, Hawkshead Lane, North Mymms, Hertfordshire AL9 7TA, UK; jbowen@rvc.ac.uk; 3 Department of Psychiatry and Forensic Medicine, Autonomous University of Barcelona, 08193 Bellaterra, Spain; jaumefatjo@gmail.com

**Keywords:** behaviour, dog, early experiences, maternal care, mother-puppy interactions, puppy

## Abstract

**Simple Summary:**

Maternal care in dogs and its impact on the behavioural development of puppies has become the subject of growing research interest. In order to determine the effects of maternal care on the behaviour of family dog puppies, maternal care during the first three weeks after birth was observed in 12 litters of puppies reared in a home environment (72 puppies). The behavioural responses of the puppies were assessed at two months of age, in an arena and an isolation test. In both tests, the amount of maternal care was found to be positively associated with some stress behaviours and, in the arena test, also with the puppies’ interest in an unfamiliar person who was present during the test. These behaviours are similar to those observed during the separation of other young mammals from an attachment figure with which they have a high quality of bond. Amount of maternal care was also found to be negatively associated with exploration and play. The difference in results between the present study and our previous study involving laboratory dogs reared in relative social isolation suggests that the developmental trajectory of puppies is influenced by a combination of maternal behaviour and social and physical environmental enrichment.

**Abstract:**

Maternal care has been shown to affect the development of the brain, behaviour, social skills and emotional systems of the young of many mammalian species including dogs. The aim of the present study was to determine the effects of maternal care on the behavioural responses of family dog puppies towards environmental and social stimuli. In order to do this, maternal care (licking puppy’s ano-genital area, licking the puppy, nursing and mother-puppy contact) during the first three weeks after birth was assessed in 12 litters of domestic dog puppies reared in home environments (total = 72 puppies). The behavioural responses of puppies were assessed in an arena and an isolation test, which were performed when the puppies were two-month old. Data were analysed using principal components analysis and projection to latent structures regression. A systematic relationship was found between maternal care and behaviour in both tests. In the arena test, maternal care was found to be positively associated with approach to the stranger, attention oriented to the stranger, time spent near the enclosure, yawning, whining and yelping (R^2^Y = 0.613, p = 8.2 × 10^−9^). Amount of maternal care was negatively associated with the number of squares crossed and the time spent individually playing with the rope. In the isolation test, the amount of maternal care was positively associated with standing posture, paw lifting, and howling, and it was negatively associated with yawning, lying down and nose licking (R^2^Y = 0.507, p = 0.000626). These results suggest that the amount of maternal care received during early life influences the pattern of behavioural responses and coping strategies of puppies at two-months of age. On the basis of these findings it could be speculated that early maternal care contributes to adaption to the environment in which family puppies are developing, with particular regard to social relationships with people.

## 1. Introduction

Maternal care plays a crucial role in the life of the offspring; in most mammals it ensures neonatal survival and represents the main source of stimuli in the early postnatal environment. Its influence on neuro-behavioural development has been well documented in a variety of species [1,2,3] including rodents [4,5,6,7], non-human primates [8,9], and humans [10,11]. 

In mice and rats there is a connection between maternal grooming and ano-genital licking received by pups in the early postnatal period, and their later behavioural patterns [12], including those related to stress responses, fear and anxiety [1,13]. Highly responsive maternal behaviour is known to promote a stress neurobiology that is less reactive as well as more resilient to challenge. The mechanisms are highly specific and involve relatively permanent modifications of DNA that control the expression of glucocorticoid receptors [14]. 

In monkeys, such as the Geoffroy’s marmoset [9] and Rhesus macaque [15], low quality or abusive maternal care during the early postnatal period produces more pronounced hypothalamic-pituitary adrenal (HPA) responses to environmental stressors throughout development and into adulthood, compared with animals reared with higher quality early maternal care. 

In human infants, sensitive and responsive maternal caregiving has an equivalent role in buffering the HPA system [16,17], and a higher quality of maternal behaviour has been found to predict a better cortisol recovery after a mildly stressful event in three-month old infants [18]. 

With respect to domestic dogs, the study of maternal behaviour has previously received comparatively little attention [3]. Rheingold’s landmark text on maternal behaviour in dogs [19] has only recently been followed up by other peer reviewed works in this area [3,20,21,22,23,24,25]. With regard to the effects of maternal care on the behavioural development of puppies, and on the subsequent behaviour of adult dogs, only few works based on observational methods have been published [26,27,28]. Results have also been inconsistent. Foyer and colleagues [26] found that, in female military German shepherd dogs, there was individual variation in the expression of maternal behaviour. They also found that mother-offspring interactions were associated with individual differences in physical and social engagement, and aggression, in the offspring. Guardini et al. [27] studied the effects of morning maternal care on 8-week-old puppies living in standardised rearing conditions with a low level of socialisation towards people. This study showed that, as previously found in rodents (see [6,7]), the quantity of maternal care received by puppies reared in standardised conditions during the first three weeks of life mediates a set of responses which allows the individual to cope with stressful situations and to better adapt to the environment. Similarly, Tiira & Lohi [29] found, through the use of a questionnaire administered to dog-owners, that fearfulness in family dogs was associated with poorer maternal care during puppyhood. However, in contradiction to the findings of Foyer et al. [26] and Guardini et al. [27], Bray et al. [28] found that increased maternal behaviour was positively associated with undesirable anxiety-related behaviours and performance in young adult dogs.

To our knowledge, the effects of maternal care on the response of family reared puppies towards unfamiliar environmental and social stimuli has not previously been investigated. This was the focus of the present study. 

The offspring-directed maternal behaviour of 12 mothers, from a range of breeds, was recorded during the first three weeks after the birth of the puppies. The behaviour of these puppies was then assessed at two months of age in two potentially stressful situations (arena and isolation tests).

In a previous study we applied the same methodology to a population of laboratory dogs [27]. In that study it was found that a higher level of maternal care was associated with more exploratory and fewer anxiety related behaviours during the isolation test. No systematic relationship was found between maternal care and arena test behaviour. However, human socialisation and external stimulation provided to laboratory puppies were significantly lower than for family reared puppies.

We therefore hypothesised that family puppies would behave differently from laboratory puppies in those situations in which social (unfamiliar person) and inanimate stimuli (toys) were present, as was the case in the arena test. For the isolation test, we expected results similar to those in our previous study [27].

## 2. Materials and Methods 

### 2.1. Subjects

Twelve litters (72 puppies) from different breeds and mixed-breeds, living with their mothers in home environments, participated in the study. Litters were recruited from Italian professional and non-professional dog breeders collaborating with the Department of Veterinary Science—University of Pisa (Italy). The characteristics of the mothers and puppies included in the study are reported in Table 1.

### 2.2. Analysis of Maternal Care

Every day, from day 1 to 21 after birth, a 15-min video of each mother with her puppies was recorded in the morning, starting when the mother returned to the whelping box after her first morning urination and/or defecation. Puppies were identified using individual coloured satin ribbons, which remained on the puppy throughout the 21-day period. 

Two tripod-mounted video-cameras were used to cover the whole whelping box. 

To assess maternal care given to each puppy, a list of behaviours from Guardini et al. [23] was used. Behaviours observed included: mother-puppy physical contact (later referred as contact), licking the ano-genital area (later referred as licking-ag), licking other parts of the puppies’ body (later referred as licking), and nursing. For each behaviour included in the list, the interaction within each specific mother-puppy dyad was analysed.

Every video was viewed multiple times, each time focussing on a specific mother-puppy dyad. Continuous sampling was used to record each type of behaviour in each video, and the duration (in seconds) of each analysed behaviour was recorded. Behaviours were not mutually exclusive. 

### 2.3. Behavioural Tests for Puppies

At 58–60 days of age, each puppy was subjected to two behavioural tests on the same day; first the arena test and then, after a break of 1–3 h, the isolation test (both previously described in Guardini et al. [27]). In the arena test, the puppies were individually placed in an enclosed unfamiliar area for 5 min with an unfamiliar female person. The stranger remained seated in a neutral pose and ignored the puppy. Four different toys were placed in the arena for the animal to explore (for the detailed arena test protocol see Guardini et al. [27]). The floor of the arena was marked into twenty-five 60 cm squares with a central circle of 1.60 m in diameter. There were minor differences in the arena used in the present study compared with the original study [27]; the arena was a 3.0 m × 3.0 m square instead of a 3.6 m × 2.2 m rectangle, and it was enclosed on all four sides by a metal fence instead of being enclosed on three sides by the walls of the test room and on the remaining side by a metal fence. 

To assess the behaviour of each puppy in the arena, five groups of behaviours were analysed: non-social behaviours, vocalisation, stress behaviours, social behaviours and “other” behaviours. For the complete description and definitions of the behaviours analysed see Guardini et al. [27]. Due to the changes in the type of enclosure used, the word “wall” was removed from the description of the following non-social behaviours: exploration, near the enclosure, behaviours oriented to the enclosure, and attention oriented outside the enclosure.

In the isolation test, each puppy was placed inside an enclosure in an unfamiliar environment where he/she remained alone for 3 min (for the detailed isolation test protocol see Guardini et al. [27]).

To assess the behaviour of each individual puppy in the isolation test, a list of four groups of behaviours was used: behavioural signs of stress, vocalisation, non-social behaviours and “other” behaviours. For the complete description and definitions of the behaviours analysed see Guardini et al. [27]. 

In both behavioural tests, the puppies were video recorded using two video-cameras, which covered the whole area of the tests.

The duration (in seconds) of each behaviour from the two lists was measured. The number of entrances in the central circle and number of squares crossed were also recorded. 

Barking, howling and growling were grouped for the statistical analysis in the arena test.

Being an observational study, ethical approval from within the University of Pisa was sufficient (approval from the National Ethical Committee was not required). 

### 2.4. Statistical Analysis

Multivariate analyses of mean-centred and unit-variance scaled data (principal components analysis—PCA), and projection to latent structures analysis (PLS) were performed using SIMCA-P+ 12^®^ software (MKS Data Analytics Solutions; Umeå, Sweden). No variables required transformation to reduce skewness. All models were created using the correlation matrix. The significance of PLS models was calculated using the analysis of variance of the cross-validated residuals (CV-ANOVA; [30]). OPLS (projection to latent structures with an integrated orthogonal signal correction filter) was used when PLS produced a model with two predictive components. Orthogonal signal correction is a method of rejecting systematic variance in X (behavioural test data) that is not correlated to Y (maternal care score), and thereby produces models with a single predictive component. The purpose of PLS (and OPLS) is to identify whether there is any systematic relationship between the X and Y variables. When validating such models, the key parameters are R^2^Y and Q^2^ (CV-ANOVA p value is also used to confirm significance). R^2^Y provides a measure of the proportion of variance in Y that can be explained by a linear combination of X variables. As with R^2^ values generated by PCA, the percentage of variance explained can be calculated by multiplying R^2^Y by 100. Q^2^ is an indication of the vulnerability of a model to the removal data or observations. Ideally, Q^2^ should be a minimum of half of the value of R^2^Y. Low values of Q^2^ indicate that a model has been unduly influenced by a small number of observations or variables, without which the model would collapse.

To produce a single measure of maternal care that could be used as Y in our multivariate models, first the mean daily amount was calculated for each of the four behaviours analysed in the videos (contact, licking, licking-ag and nursing) for each mother-pup dyad. From these, a simple score of total maternal care could have been calculated, but this would have assumed identical weighting for each of the four maternal care variables. Instead we used PCA as a method of dimension reduction, to generate scores for maternal care that better represented the data. Data was first mean centred and unit variance scaled. PCA, using the correlation matrix and without rotation, generated a model with a single principal component (R^2^ = 0.544, Q^2^ = 0.236, KMO measure of sampling accuracy = 0.549, Bartlett’s test of sphericity was passed with p < 0.0001). The number of final principal components (PCs) was determined by a combination of eigenvalue (greater than 1), and fall off of Q^2^ with the addition of further PCs. A decrease in Q^2^ indicates that the addition of further PCs will reduce the robustness of the model, even if R^2^ is increased. A summary of loadings is included in Table 2. Scores from this model were used to summarise the amount of maternal care as a single value for each mother-pup dyad (hereafter referred to as the “maternal care score”). This maternal care score was used as the regression (Y) variable for subsequent multivariate models that investigated the relationship between maternal behaviour and performance in the arena and isolation tests.

A cross-validated PLS regression model was constructed using the maternal care score as the regression variable (Y) and the arena test data as the set of X variables. A second cross-validated PLS regression model was constructed using the maternal care score as the regression variable (Y) and the isolation test data as the set of X variables.

## 3. Results

**Figure 1:**
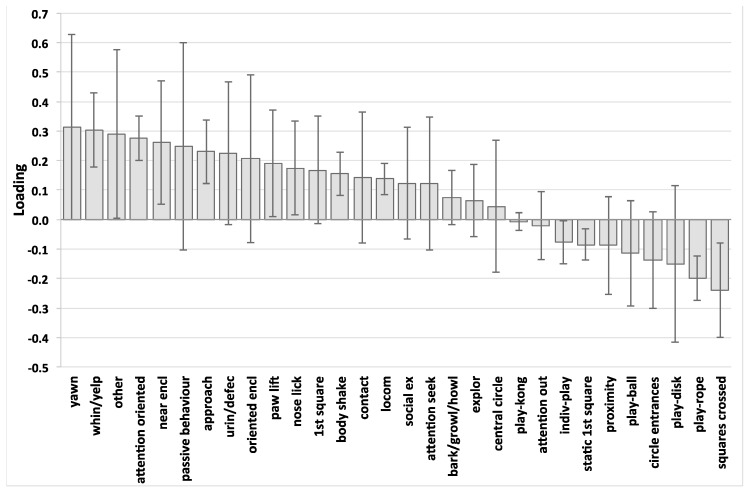
Loadings bar chart of projection to latent structures analysis (PLS) of arena test variables versus maternal care principal components analysis (PCA) score. Positive loadings (upward pointing bars) indicate the puppy behaviours that are positively associated with maternal care score, and negative loadings (downward pointing bars) indicate the puppy behaviours that are negatively associated with maternal care score. Height of bars is an index of the strength of that association. However, the chart should be viewed and interpreted as a pattern. Whiskers indicate 95% confidence interval. Legend: yawn (Yawning), whin/yelp (Whining/Yelping), other (Other behaviours), attention oriented (Attention oriented to the stranger), near encl. (Near the enclosure), passive behaviour (Passive behaviour), approach (Approach), urin/defec (Urination and/or defecation), orientated encl (Behaviours orientated to the enclosure), paw lift (Paw lifting), nose lick (Nose licking), 1st square (Puppy in the 1st square), body shake (Body shaking), contact (Physical contact with the stranger), locom (Locomotion), social ex (Social exploration), attention seek (Attention seeking), bark/growl/howl (Barking, Growling, Howling), explor (Exploration), central circle (Puppy in the central circle), play-kong (Individual play-Kong), attention out (Attention oriented outdoor the enclosure), indiv-play (Individual play), static 1st square (Static in the 1st square), proximity (Proximity), play-ball (Individual play-ball), circle entrances (Number of entrances in the central circle), play-disk (Individual play-plastic disk), play-rope (Individual play-rope), squares crossed (Number of squares crossed).

**Figure 2:**
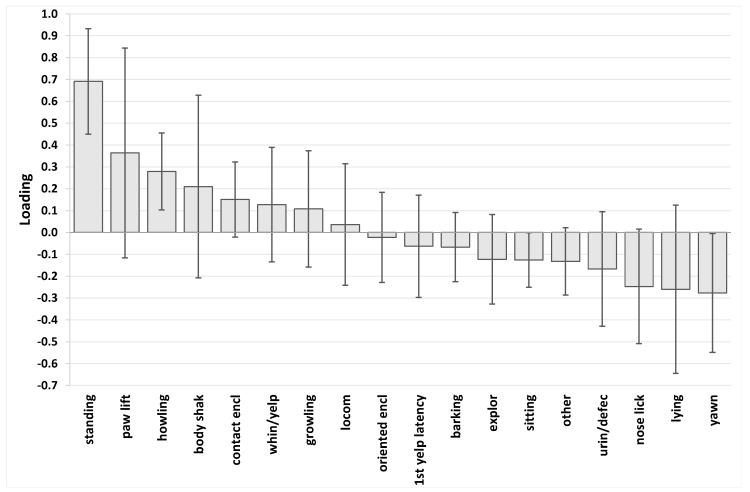
Loadings bar chart of OPLS of isolation test variables versus maternal care PCA score. Positive loadings (upward pointing bars) indicate the puppy behaviours that are positively associated with maternal care score, and negative loadings (downward pointing bars) indicate the puppy behaviours that are negatively associated with maternal care score. Height of bars is an index of the strength of that association. However, the chart should be viewed and interpreted as a pattern. Whiskers indicate 95% confidence interval. Legend: standing (Standing), paw lift (Paw lifting), howling (Howling), body shak (Body shaking), contact encl (In contact with the enclosure), whin/yelp (Whining/Yelping), growling (Growling), locom (Locomotion), orientated encl (Behaviours orientated to the enclosure), 1st yelp latency (Latency to the first yelp/whine), barking (Barking), explor (Exploration), sitting (Sitting), other (Other behaviours), urin/defec (Urination and/or defecation), nose lick (Nose licking), lying (Lying), yawn (Yawn).

The PLS of the arena test data produced a model with a single predictive component (R^2^Y = 0.613, Q^2^ = 0.462, CV-ANOVA p = 8.2 × 10^−9^). This indicates a strong and highly significant systematic relationship between individual puppy behaviours in the arena test and maternal care (see Figure 1 for a loadings plot for this PLS model). Both loadings charts (Figure 1 for the arena test and Figure 2 for the isolation test) should be read as illustrations of the patterns of associations between the X and Y variables in the two models, with level of contribution being broadly related to loading magnitude. However, although it is useful to mention key variables when attempting to interpret the meaning of the models, neither the magnitude of individual loadings, nor the confidence intervals associated with them, should be taken out of context; the overall pattern of behaviour is the key result. Confidence intervals are included for completeness, but overall model evaluation should be based only on R^2^Y, Q^2^ and CV-ANOVA. 

In the arena test, the strongest positive loadings (variables positively associated with maternal care score) were Yawning, Whining/Yelping, Near the enclosure, Attention oriented to the stranger and Approach. The strongest negative loadings (variables negatively associated with maternal care score) were Number of squares crossed and Individual play-rope. However, rather than focus on the individual loadings, the key finding is that there is a systematic relationship between maternal care and the pattern of the puppies’ arena test behaviour, characterised by stress signs, approach and attention oriented to the stranger, time spent near the enclosure, and a lack of exploratory or play behaviour.

The PLS of the isolation test data produced a model with two predictive components (R^2^Y = 0.507, Q^2^ = 0.287, CV-ANOVA p = 0.000562). Since it is hard to interpret PLS models that have more than a single predictive component, orthogonal signal correction was used to reject systematic variance in X that was uncorrelated with Y. The resultant cross-validated OPLS model included a single orthogonal component (representing variance in X that is not correlated with Y), and a single predictive component (representing variance in X that is correlated with Y) and there was minimal effect on model quality compared with PLS (R^2^Y = 0.507, Q^2^ = 0.282, CV-ANOVA p = 0.000626). This indicates a significant systematic association between individual puppy behaviours in the isolation test and maternal care (see Figure 2 for a loadings plot for this OPLS model). 

In the isolation test, the strongest positive loadings (variables positively associated with maternal care score) were Standing, Paw lifting and Howling, although the value for Paw lifting was undermined by its very wide confidence interval. The strongest negative loadings (variables negatively associated with maternal care score) were Lying, Yawn, and Nose licking. Again, rather than focus on the individual loadings, the key finding is that there is a systematic relationship between maternal care and the pattern of isolation test behaviour, characterised by stress signs, passive standing, paw lifting and not resting.

## 4. Discussion

Early life experiences are known to shape the behavioural development of animals. Previous studies in different species have demonstrated that maternal care plays a key role in the offspring’s ontogeny (e.g., [1,3,31]), and such results have also been found in domestic dogs [26,27,28]. However, to our knowledge, this is the first study investigating the effect of maternal care on the behaviour of two-month old puppies that have been reared in a family environment. 

In the present study, the use of multivariate statistical methods (PLS and OPLS) identified statistically significant systematic relationships between maternal care and behaviour in both the arena and isolation tests. This suggests that the amount of maternal care (in terms of nursing, body licking, ano-genital licking, and physical contact between mother and puppy) received during the 21 days after birth has an effect on the pattern of behaviour of family-reared puppies when they are faced with unfamiliar environmental and social stimuli at two months of age. As opposed to individual correlations between variables, multivariate relationships of this kind indicate the presence of patterns of altered behaviours that point to an underlying latent process. 

The puppies’ behaviour in the arena test was systematically associated with the amount of maternal care received during the first 21 days of life (as summarised by the maternal care score). The strongest positive loadings (variables positively associated with maternal care score) within that model were certain stress behaviours (increase of yawning and whining/yelping), the time spent near the enclosure and the puppies’ interest in the unfamiliar human figure (increased orientation and approach). In contrast, the strongest negative loadings (variables negatively associated with maternal care score) were the puppies’ activity, interest in his/her surroundings and the inanimate stimuli (reduced number of crossed squares and time spent playing with the rope). Increased maternal care was associated with increased social interest shown by family reared puppies towards the stranger in the arena. The pattern of positive loadings found in the arena test in the present study are comparable with those behaviours observed during the activation of the attachment system when a young mammal is separated from its attachment figure in an unfamiliar environment, as observed in the Ainsworth Strange Situation Test (ASST, [10]), which is a paradigm created for the assessment of the attachment bond between children and their caregivers. The stress signs and the vocalisations emitted by the family reared puppies in the arena test are comparable with the protest behaviours children show when separated from their mothers [10], which have also been documented in infant monkeys [32,33], lambs [34], and domestic dog puppies [35,36,37]. Remaining near the enclosure boundary could be interpreted as an attempt to regain proximity to the attachment figure. This behaviour is similar to “stay by” and “be oriented to the door” in children [10] and adult dogs [38] when tested in the ASST, and to the attempts to break the barriers separating infant rhesus monkeys from their mothers [39]. It seems that the more maternal care a family reared puppy receives during early life, the more distress he/she shows during separation, and the more orientated he/she is to reunification with the attachment figure or obtaining social support from another social partner (in this case the stranger in the arena). This is likely to be due to the higher quality of the attachment bond between the mother and the offspring. The puppies stay close to the unfamiliar person, and attempt to seek attention in order to obtain comfort and support in the absence of the attachment figure. The same tendency to seek contact and support from an unfamiliar individual, in the absence of a familiar individual or attachment figure, has been previously observed in children [11], young chimpanzees [40], and adult dogs [38,41,42]. In the present study, the level of maternal care seems to influence the puppy’s motivation and/or strategy when faced with a stressful situation, in this case seeking support and comfort from an unfamiliar human being. The puppies’ attempt to buffer stress through social contact with a human is supported by the work of Pettijhon et al. [43], in which it was observed that the human figure was very effective in alleviating separation-induced distress vocalisation in young puppies, especially if the person behaved actively rather than passively. In the current study, the stranger was instructed to behave passively, and therefore any relief from separation distress that the puppies might have obtained from contact with that person would only have been partial. Stress behaviours observed in the arena test in the present study might therefore also have been influenced by this failed distress buffering. 

The fact that maternal care has an impact upon the puppies’ interest in a social stimulus is an important finding, and is in agreement with studies in other mammalian species (e.g., rats: [44,45]; prairie voles: [46]; rhesus macaques: [47,48], including humans [49] and adult military German shepherds dogs [26]. With regard to an underlying biological basis for this effect on social behaviour, it has been observed that the quantity and the quality of maternal care influences oxytocin receptor density in the brain in rodents [2,44], humans [31,50], in rhesus monkeys [51], as well as various neurochemicals which including vasopressin, prolactin, catecholamines, endogenous opioids, adrenocorticotropic hormone, and gamma-aminobutyric acid [52]. Although there is a lack of knowledge about the impact of maternal care upon oxytocin levels in *Canis Familiaris* puppies, it is likely that a similar mechanism is present in this species. 

The tendency to seek security from strangers is potentially very important for domestic dog puppies, who have to forge a close relationship with humans when they are taken away from their mothers and introduced into a new home. A positive attitude towards human strangers at two months of age, which is the usual time of adoption, may in fact make the separation from the mother and the establishment of a bond with the new owners easier. Further research is needed in order to more deeply understand the link between the amount of maternal care, oxytocin levels and puppies’ attitude towards people. 

In our previous study [27], the same protocol was used to investigate the influence of morning maternal care on two-month old puppies reared in standardised conditions with limited exposure to human beings. The findings were quite different: In that study, no significant systematic relationship was found between maternal care score and the puppies’ behaviour in the arena test. This difference may be due to general effects of maternal care on the puppy’s adaptability to the environment and his/her inclination to seek social support, combined with the higher level of interspecific socialisation of the family reared puppies that enabled them to regard an unfamiliar person as a potential source of security. The presence of this source of security may also have enabled the puppies to orientate their coping strategy more consistently, leading to a more systematic organisation of behaviour that was not detectable in laboratory puppies.

Contrary to the original hypothesis, higher scores for maternal care were associated with reduced interest in the environment or the objects that were present in the arena test. These findings also contradict some of the previous literature on the effects of maternal care in other species. In rodents, increased ano-genital licking and grooming by the mother is correlated with an increase in play behaviour by the offspring [31,53,54], as well as in exploratory behaviour [4,5,13]. This could, again, be explained as being a consequence of the developmental environment; puppies reared in a family are likely to be more focussed on people than on physical stimuli during the arena test. This explanation is supported by data from a previous work [27], which found that higher morning maternal care scores were associated with increased exploratory behaviour in puppies reared in an environment with low interspecific socialisation. 

The puppies’ behaviour in the isolation test was also systematically associated with the amount of maternal care received during the first 21 days of life, although this model was weaker than the arena test behaviour model. The strongest positive loadings in this model were for standing posture, paw lifting and howling. Howling is a distress vocalisation, which can be interpreted as et-epimeletic or care soliciting behaviour [35,55,56], emitted by the puppies in order to call the mother and to regain contact with her. Standing posture could be interpreted as the puppy waiting for signs of social reunification (i.e., with the mother). As in the arena test, it seems that the more maternal care a puppy received during his/her early life, the greater his/her need to regain proximity with his/her mother during separation (and hence the greater the distress observed during isolation). The important difference between the arena and the isolation test was the absence, in the latter, of any form of potential social support. When family puppies were completely alone they vocalised, but if a stranger was present, puppies tried (although unsuccessfully) to seek comfort and support from that person (otherwise they showed separation distress). It can be hypothesised that the level of maternal care influences the type of stress responses shown by family puppies in this stressful situation (absence of the mother and littermates and being in an unfamiliar environment). In the isolation test model, the strongest negative loadings were with lying, yawn, and nose licking. The latter two are subtle stress signs [57].

The initial hypothesis that we would find similar results in the isolation test to those of our previous study involving laboratory reared puppies [27] was not confirmed. In the laboratory puppies, the amount of maternal care was most strongly positively associated with engagement with the environment inside the metal fences, less destructive behaviours and reduced non-exploratory locomotion. Those results are more similar to those from the literature in rodents (see [1,4,5,6,13,58,59,60]). The difference may relate to the diversity of breeds in the present study, and the different sensory environments to which the puppies were exposed. A family home is a much more complex and stimulating environment than a laboratory kennel, and the family reared puppies had far greater contact with people. It is possible that family reared puppies found the arena environment less engaging because they were already used to more complex and stimulating environments. The opposite could be said for the laboratory puppies. 

Another potentially influential factor in this study is litter size. Litter size can have an effect on the amount of maternal behaviour available to individual puppies; when the mother’s time and energy are divided between members of a larger litter, we might expect individual mother-pup interactions to be reduced. Unfortunately, due to the small number of litters and relatively uniform litter size in the present study, it was not possible to study the effect of litter size. Given the confounding effects of environmental variation within and between homes, if the effect of litter size were to be investigated, it would probably be easier to identify an effect in laboratory reared pups with a standardised environment.

The results of the present study are likely to be more applicable to dogs that live in an enriched environment that includes social stimuli, as is the experience of most companion and working dogs. Interestingly, Bray et al. [28] also found an association between the amount of maternal care received and anxiety behaviours in young guide dogs between 14 and 17 months of age; dogs that received higher levels of maternal care showed behaviours related to stress and anxiety when isolated in an empty room (e.g., high activity and short latency to vocalisations when presented with a novel object). Bray et al. [28] took into account the nursing style of the mothers and found that mothers whose nursing style (vertical nursing) required greater effort by puppies were more likely to produce offspring that were successful in cognitive and temperament tasks, whereas mothers that used a ventral nursing style that required less effort from the puppies were more likely to produce offspring that failed. The authors proposed that there might be benefits from a moderate amount of stress during early life: vertical nursing style may provide opportunities for puppies to cope with small challenges which are beneficial and adaptive in the long term, enhancing arousal regulation and resilience. 

Future research may clarify how maternal factors, the social environment and also genetic effects contribute to the expression of pet dogs’ behaviour in different phases of development, from puppyhood to adulthood.

## 5. Conclusions

The quantity of maternal care received by family puppies in early life influences their behaviour in an unknown environment, in the presence and in the absence of a stranger. Specifically, maternal care was associated not only with increased interest in a human stranger, but also with increased display of separation-related stress. Although these findings disagree with some previous studies of the development of animals, including our own study involving puppies reared in conditions of low socialisation with humans, they help to provide further insight into the subtle interaction between maternal behaviour and environmental exposure during development.

## Figures and Tables

**Table 1:** Characteristics of the 12 litters included in the study.

Litter	Breed	Mother’s Age (Months)	Parity	N° Puppies	Males	Females	Provenance
1	Weimaraner	18	1	5	3	2	professional breeder
2	Belgian Shepherd Groenendal	72	1	4	4	0	professional breeder
3	Cross breed	24	1	3	2	1	not professional breeder
4	Belgian Shepherd Groenendal	72	3	7	4	3	professional breeder
5	Short Haired Dachshund	60	3	5	2	3	professional breeder
6	Belgian Shepherd Groenendal	84	2	5	4	1	professional breeder
7	German Shepherd	72	3	7	2	5	not professional breeder
8	Labrador Retriever	24	1	7	5	2	not professional breeder
9	Boxer	84	3	9	1	8	professional breeder
10	Border Collie	24	1	6	4	2	not professional breeder
11	Deutsch Drathaar	18	1	5	3	2	professional breeder
12	Boxer	32	1	8	3	5	not professional breeder
	mean ± standard deviation	48.7 ± 27.4	1.7 ± 1.0	5.9 ± 1.7	3.0 ± 1.2	2.8 ± 2.2	

**Table 2:** Loadings of the principal component.

Var ID (Primary)	Loading
MEAN__Puppy_contact	0.635562
MEAN__Puppy_nursing	0.622256
MEAN__Puppy_licking	−0.047369
MEAN__Puppy_licking ag	0.454549
